# The development of a cSMART-based integrated model for hepatocellular carcinoma diagnosis

**DOI:** 10.1186/s13045-022-01396-z

**Published:** 2023-01-05

**Authors:** Tong Wu, Rong Fan, Jian Bai, Zhao Yang, Yun-Song Qian, Lu-Tao Du, Chun-Ying Wang, Ying-Chao Wang, Guo-Qing Jiang, Dan Zheng, Xiao-Tang Fan, Bo Zheng, Jing-Feng Liu, Guo-Hong Deng, Feng Shen, He-Ping Hu, Yi-Nong Ye, Qing-Zheng Zhang, Jing Zhang, Yan-Hang Gao, Jie Xia, Hua-Dong Yan, Min-Feng Liang, Yan-Long Yu, Fu-Ming Sun, Yu-Jing Gao, Jian Sun, Chun-Xiu Zhong, Yin Wang, Hui Wang, Fei Kong, Jin-Ming Chen, Hao Wen, Bo-Ming Wu, Chuan-Xin Wang, Lin Wu, Jin-Lin Hou, Xiao-Long Liu, Hong-Yang Wang, Lei Chen

**Affiliations:** 1 International Cooperation Laboratory on Signal Transduction, National Center for Liver Cancer, Eastern Hepatobiliary Surgery Institute/hospital, Shanghai, 200438 People’s Republic of China; 2 Department of Radiotherapy Oncology, General Hospital of Northern Theater Command, Shenyang, 110016 People’s Republic of China; 3 Department of Infectious Diseases, State Key Laboratory of Organ Failure Research, Guangdong Provincial Key Laboratory of Viral Hepatitis Research, Nanfang Hospital, Southern Medical University, Guangzhou, 510515 People’s Republic of China; 4 Hepatology Unit, Shenzhen Hospital, Southern Medical University, Shenzhen, People’s Republic of China; 5 Berry Oncology Corporation, Beijing, 100102 People’s Republic of China; 6 Eastern Hepatobiliary Surgery Hospital, Second Military Medical University, Shanghai, 200438 People’s Republic of China; 7 Hepatology Department, Ningbo Hwamei Hospital, University of Chinese Academy of Sciences, Ningbo, 315010 People’s Republic of China; 8 Department of Clinical Laboratory, The Second Hospital, Cheeloo College of Medicine, Shandong University, 247 Beiyuan Street, Jinan, 250033 Shandong People’s Republic of China; 9 Shandong Provincial Clinical Medicine Research Center for Clinical Laboratory, Jinan, 250033 People’s Republic of China; 10 Xuzhou Infectious Diseases Hospital, Xuzhou, 221004 People’s Republic of China; 11 The United Innovation of Mengchao Hepatobiliary Technology Key Laboratory of Fujian Province, Mengchao Hepatobiliary Hospital of Fujian Medical University, Fuzhou, 350025 People’s Republic of China; 12 Department of Hepatobiliary Surgery, Clinical Medical College, Yangzhou University, Yangzhou, 225001 People’s Republic of China; 13 Department of Gastroenterology, The Central Hospital of Wuhan, Tongji Medical College, Huazhong University of Science and Technology, Wuhan, 430014 People’s Republic of China; 14 Department of Hepatology, First Affiliated Hospital of Xinjiang Medical University, Urumqi, 830000 People’s Republic of China; 15 Department of Infectious Diseases, Southwest Hospital, Third Military Medical University (Army Medical University), Chongqing, 400038 People’s Republic of China; 16 Department of Hepatic Surgery IV, Eastern Hepatobiliary Surgery Hospital, Second Military Medical University, Shanghai, 200438 People’s Republic of China; 17 Department of Hepatobiliary Medicine, Shanghai Eastern Hepatobiliary Surgery Hospital, Shanghai, 210822 People’s Republic of China; 18 The Department of Infectious Disease, The First People’s Hospital of Foshan, Foshan City, 528000 People’s Republic of China; 19 The First Hospital of Jilin University, Jilin, 130021 People’s Republic of China; 20 Chifeng Clinical Medical School of Inner, Mongolia Medical University, Chifeng, 024000 People’s Republic of China; 21 State Key Laboratory of Pathogenesis, Prevention and Treatment of High Incidence Diseases in Central Asia, First Affiliated Hospital of Xinjiang Medical University, Urumqi, 830000 People’s Republic of China; 22 Key Laboratory of Signaling Regulation and Targeting Therapy of Liver Cancer (SMMU), Ministry of Education, Shanghai, 200438 People’s Republic of China; 23 Shanghai Key Laboratory of Hepatobiliary Tumor Biology (EHBH), Shanghai, 200438 People’s Republic of China

**Keywords:** Cell-free DNA, Hepatocellular carcinoma, Mutation, Biomarker, Diagnosis

## Abstract

**Background:**

Hepatocellular carcinoma (HCC) generally arises from a background of liver cirrhosis (LC). Patients with cirrhosis and suspected HCC are recommended to undergo serum biomarker tests and imaging diagnostic evaluation. However, the performance of routine diagnostic methods in detecting early HCC remains unpromising.

**Methods:**

Here, we conducted a large-scale, multicenter study of 1675 participants including 490 healthy controls, 577 LC patients, and 608 HCC patients from nine clinical centers across nine provinces of China, profiled gene mutation signatures of cell-free DNA (cfDNA) using Circulating Single-Molecule Amplification and Resequencing Technology (cSMART) through detecting 931 mutation sites across 21 genes.

**Results:**

An integrated diagnostic model called “Combined method” was developed by combining three mutation sites and three serum biomarkers. Combined method outperformed AFP in the diagnosis of HCC, especially early HCC, with sensitivities of 81.25% for all stages and 66.67% for early HCC, respectively. Importantly, the integrated model exhibited high accuracy in differentiating AFP-negative, AFP-L3-negative, and PIVKA-II-negative HCCs from LCs.

**Supplementary Information:**

The online version contains supplementary material available at 10.1186/s13045-022-01396-z.

## To the editor,

Hepatocellular carcinoma (HCC) is the sixth most common cancer and ranks the fourth in cancer mortality worldwide, and patients with liver cirrhosis (LC) are at high risk of HCC [1, 2]. Constantly elevated levels of alpha-fetoprotein (AFP) and other serum biomarkers including AFP-L3 and PIVKA-II generally indicate development of HCC; however, the performance of these biomarkers as diagnostic models for early HCC remains unpromising [3].

The utility of cancer-associated aberrations including genic mutations in cell-free DNA (cfDNA) for cancer detection is a global research hot spot [4, 5]. Circulating Single-Molecule Amplification and Resequencing Technology (cSMART) is a detection platform that can simultaneously detect and quantitate multiple plasma DNA variants based on next-generation sequencing [6, 7]. A total of 1702 individuals (healthy cohort, LC cohort, and HCC cohort) from nine clinical sites across China were enrolled from June 2018 through January 2019 in this study. In HCC cohort, 27 were excluded according to pathology diagnosis. Finally, 1675 participants (490 healthy controls, 577 LC patients, and 608 HCC patients) were randomly assigned to training/validation/test cohorts (Additional file 1: Fig S1). Detail information of these participants is shown in Additional files 1, 2: Tables S1–S8. 10 mL peripheral blood was provided from each individual for cSMART test at enrollment time.

**Fig. 1:**
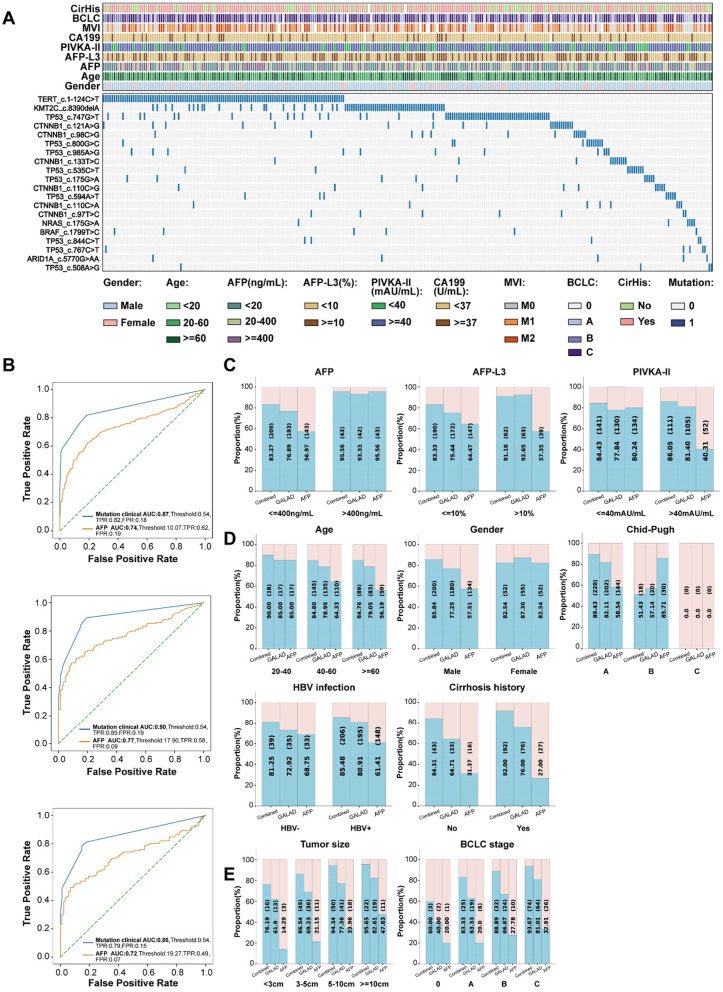
Combined method holds a strong value in diagnosis of HCC. **a** Basic information (age, gender), cirrhosis background, tumor serological biomarkers (CA199, PIVKA-II, AFP-L3, AFP), and HCC related parameters (MVI and BCLC stages) of all HCC samples with positive mutations at the top 20 high-frequency mutation sites. CA199: carbohydrate antigen199; MVI: microvascular invasion; and BCLC: Barcelona clinic liver cancer. **b** ROC curves of Combined method and AFP for HCC patients versus LC patients in the training, test, and validation cohorts. **c** Proportions of positive and negative calling by Combined method, GALAD, and AFP in all participants with different AFP, AFP-L3, and PIVKA-II levels in test cohort. **d** Proportions of positive and negative calling by Combined method, GALAD, and AFP in all participants with different age, gender, Child–Pugh stages, HBV infection status, and cirrhosis history in test cohort. **e** Proportions of positive and negative calling by Combined method, GALAD, and AFP in HCC patients with different tumor sizes and BCLC stages in test cohort

We first constructed negative background pool using cfDNA samples from 490 healthy individuals. To explore the feature of cfDNA mutations in HCC and LC, 931 regions among 21 genes of 608 HCCs and 577 LCs were detected by cSMART. Top 20 gene mutation sites with high mutation frequency are detailed in Additional files 3, 4: Tables S9–S12. The overall mutation ratio of cfDNA in HCC was significantly higher than that in LC (Fig. 1a and Additional file 1: Fig. S2). Then, detected mutations were minimized and finally three mutation sites located in different regions of gene TERT, TP53, and CTNNB1 were screened out to be further analysis. The performance of the single mutation gene site in the diagnosis of HCC is shown in Additional file 1: Table S13. A gradual increasing trend in variant allele frequency (VAF) at HCC-specific mutation sites from early HCC (BALC 0/A) to advanced HCC (BCLC C) was identified (Additional file 1: Fig. S3), proving that cSMART was sensitive for quantification of low-copy number DNA in plasma and could accurately reflect the tumor mutational burden.

By integrating three mutations of cfDNA and three serum biomarkers (AFP, AFP-L3, and PIVKA-II), Combined method was developed for diagnosis of HCC. AFP, the most commonly used biomarker, could detect 43 of 151 HCCs in test cohort, and 26 of 112 HCCs in validation cohort at the cutoff value of 400 ng/mL, and achieved diagnostic sensitivity of 56.29%/48.21% at specificity of 91.03%/93.18% in test cohort or validation cohort at 20 ng/mL cutoff value. Combined method showed better performance compared with AFP, detecting 135 of 151 HCCs with a sensitivity of 89.40% at 80.69% specificity in test cohort. More, the sensitivities of this model to detect HCC at BCLC 0 and A were 60.00% and 83.87%, respectively (Table 1). The same conclusion could also be drawn from the data of the independent validation cohort (Table 1). Receiver operating characteristic (ROC) curve further corroborated that this cfDNA-based integrated diagnostic model was significantly superior to AFP in the diagnosis of HCC (Fig. 1b).

**Table 1:** Performance of Combined method in the diagnosis of HCC

	Test cohort (151HCC + 145LC)				Validation cohort (112HCC + 88LC)			
	N	Sensitivity (95% CI)	Specificity (95% CI)	Accuracy (95% CI)	N	Sensitivity (95% CI)	Specificity (95% CI)	Accuracy (95% CI)
*Combined method*								
BCLC 0-C	151	89.40 (83.09–93.63)	80.69 (73.13–86.58)	85.14 (81.08–89.19)	112	81.25 (72.54–87.77)	81.82 (71.85–88.94)	81.5 (76.12–86.88)
BCLC 0 + A	36	80.56 (63.43–91.20)	80.69 (73.13–86.58)	80.66 (74.91–86.42)	27	66.67 (46.02–82.76)	81.82 (71.85–88.94)	78.26 (70.72–85.80)
BCLC 0	5	60 (17.04–92.74)	80.69 (73.13–86.58)	80 (73.60–86.40)	7	42.86 (11.81–79.76)	81.82 (71.85–88.94)	78.95 (70.75–87.15)
BCLC A	31	83.87 (65.53–93.90)	80.69 (73.13–86.58)	81.25 (75.48–87.02)	20	75.00 (50.59–90.41)	81.82 (71.85–88.94)	80.56 (73.09–88.02)
BCLC B	36	88.89 (73.00–96.38)	80.69 (73.13–86.58)	82.32 (76.76–87.88)	28	82.14 (62.42–93.23)	81.82 (71.85–88.94)	81.90 (74.89–88.90)
BCLC C	79	93.67 (85.21–97.65)	80.69 (73.13–86.58)	85.27 (80.63–89.91)	57	87.72 (75.71–94.51)	81.82 (71.85–88.94)	84.14 (78.19–90.08)
*AFP (cutoff value: 400ng/mL)*								
BCLC 0-C	151	28.48 (21.58–36.49)	98.62 (94.60–99.76)	62.84 (57.33–68.34)	112	23.21 (15.98–32.32)	98.86 (92.95–99.94)	56.5 (49.63–63.37)
BCLC 0 + A	36	19.44 (8.80–36.57)	98.62 (94.60–99.76)	82.87 (77.38–88.36)	27	7.41 (1.29–25.75)	98.86 (92.95–99.94)	77.39 (69.75–85.04)
BCLC 0	5	20.00 (1.05–70.12)	98.62 (94.60–99.76)	96 (92.86–99.14)	7	0.00 (0.00–0.00)	98.86 (92.95–99.94)	91.58 (85.99–97.16)
BCLC A	31	19.35 (8.12–38.06)	98.62 (94.60–99.76)	84.66 (79.33–89.98)	20	10 (1.75–33.13)	98.86 (92.95–99.94)	82.41 (75.23–89.59)
BCLC B	36	27.78 (14.79–45.43)	98.62 (94.60–99.76)	84.53 (79.26–89.80)	28	25 (11.43–45.22)	98.86 (92.95–99.94)	81.03 (73.90–88.17)
BCLC C	79	32.91 (23.00–44.50)	98.62 (94.60–99.76)	75.45 (69.81–81.08)	57	29.82 (18.80–43.57)	98.86 (92.95–99.94)	71.72 (64.39–79.05)
*AFP (cutoff value: 20ng/mL)*								
BCLC 0-C	151	56.29 (47.99–64.27)	91.03 (84.85–94.95)	73.31 (68.27–78.35)	112	48.21 (38.75–57.81)	93.18 (85.19–97.20)	68 (61.54–74.46)
BCLC 0 + A	36	47.22 (30.76–64.27)	91.03 (84.85–94.95)	82.32 (76.76–87.88)	27	22.22 (9.38–42.73)	93.18 (85.19–97.20)	76.52 (68.77–84.27)
BCLC 0	5	40 (7.26–82.96)	91.03 (84.85–94.95)	89.33 (84.39–94.27)	7	14.29 (0.75–57.99)	93.18 (85.19–97.20)	87.37 (80.69–94.05)
BCLC A	31	48.39 (30.56–66.60)	91.03 (84.85–94.95)	83.52 (78.04–89.00)	20	25.00 (9.60–49.41)	93.18 (85.19–97.20)	80.56 (73.09–88.02)
BCLC B	36	50.00 (33.22–66.78)	91.03 (84.85–94.95)	82.87 (77.38–88.36)	28	39.29 (22.13–59.27)	93.18 (85.19–97.20)	80.17 (72.92–87.43)
BCLC C	79	63.29 (51.64–73.64)	91.03 (84.85–94.95)	81.25 (76.14–86.36)	57	64.91 (51.06–76.76)	93.18 (85.19–97.20)	82.07 (75.83–88.31)

Next, the accuracy of Combined method to differentiate HCC from LC was evaluated in different subgroups and compared with GALAD and AFP. In test cohort, this model could not only distinguish AFP-positive HCC from LC (accuracy: 95.56%), but also detect AFP-negative HCC who might be missed by conventional diagnostic approaches (accuracy: 83.27%). Furthermore, Combined method exhibited high accuracy for HCC diagnosis in both AFP-L3/PIVKA-II-positive and AFP-L3/PIVKA-II-negative subgroups, outperforming current commonly used biomarkers without over diagnosis (Fig. 1c). In addition, Combined method held high accuracy in diagnosis of liver tumors with any size irrespective of age, gender, Child–Pugh stage, HBV infection status, and cirrhosis history and showed much better performance in detecting early and very early HCC (accuracy: BCLC 0: 60.00%; BCLC A: 83.33%) than GALAD and AFP (Fig. 1d, e). Subsequently, the above conclusions were further confirmed in validation cohort (Additional file 1: Fig. S4).

In conclusion, we developed a retrospective phase 3 study according to the criteria for biomarker development delineated by Pepe et al., identified the unique cfDNA hotspot mutation signature of HCC, and constructed Combined method based on three mutation sites and three serum biomarkers [8]. Combined method has fixed indicators and simple detection process, outperforming conventional approaches in the diagnosis of HCC, especially early HCC, in a noninvasive way. Our model holds great potentials to be incorporated into current clinical care considering its cost-effectiveness and practicality, which is expected to improve the outcomes for HCC patients missed by traditional methods in the future.

## Supplementary Information

**Additional file 1:** Methods. Supplementary figures and legends. **Table S1:** Basic information of enrolled patients. **Table S2:** Brief summary of all participants. **Table S13:** Performance of single mutation site in the diagnosis of HCC.

**Additional file 2: Table S3:** Detail information of 345 HCC patients in training cohort. **Table S4:** Detail information of 344 LC patients in training cohort. **Table S5:** Detail information of 151 HCC patients in test cohort. **Table S6:** Detail information of 145 LC patients in test cohort. **Table S7:** Detail information of 112 HCC patients in validation cohort. **Table S8:** Detail information of 88 LC patients in validation cohort.

**Additional file 3: Table S9:** Information of top 20 gene mutation sites.

**Additional file 4: Table S10:** Analysis of top 20 gene mutations in paired cfDNA and tissue samples of HCC patients. **Table S11:** Analysis of top 20 gene mutations in paired cfDNA and tissue samples of LC patients. **Table S12:** Analysis of top 20 gene mutations in paired cfDNA and tissue samples of healthy controls.

## Data Availability

The data that support the findings of this study have been deposited into CNGB Sequence Archive (CNSA) of China National GeneBank DataBase (CNGBdb) with accession number CNP0003313. To access this data, please contact the corresponding author (Hong-Yang Wang, hywangk@vip.sina.com).

## Abbreviations

HCC: Hepatocellular carcinoma
LC: Liver cirrhosis
cfDNA: Cell-free DNA
cSMART: Circulating Single-Molecule Amplification and Resequencing Technology
AFP: Alpha-fetoprotein
CSCO: Chinese Society of Clinical Oncology
VAF: Variant allele frequency
ROC: Receiver operating characteristic
PCR: Polymerase chain reaction
AASLD: American Association for the Study of Liver Diseases
XGBoost: Extreme Gradient Boosting
