# Supplementary material for: The development of a cSMART-based integrated model for hepatocellular carcinoma diagnosis
Source: J Hematol Oncol. 2023 Jan 5;16:1. doi: 10.1186/s13045-022-01396-z (PMC9814336; doi:10.1186/s13045-022-01396-z)
Supplement: Supplementary file 1 — Additional file 1: Methods. Supplementary figures and legends. Table S1: Basic information of enrolled patients. Table S2: Brief summary of all participants. Table S13: Performance of single mutation site in the diagnosis of HCC. [file 13045_2022_1396_MOESM1_ESM.docx]

**The development of a** **cSMART-based integrated model for hepatocellular carcinoma diagnosis**

# Methods

**Study design**

490 healthy participants and 1185 individuals pre-diagnosed with LC (n=577) or HCC (n=608) from nine clinical sites across China through 9^th^ March, 2018 to 22^th^ January, 2019 were recruited in this retrospective cohort study. The clinical sites are: (1)Ningbo No.2 Hospital (Zhejiang Province), (2)Eastern Hepatobiliary Surgery Hospital (Shanghai), (3)Mengchao Hepatobiliary Hospital (Fujian Province), (4)the first affiliated hospital of Xinjiang Medical University (Xinjiang Province), (5)the second affiliated hospital of Shandong University (Shandong Province), (6)Nanfang Hospital, Southern Medical University (Guangdong Province), (7)Southwest Hospital (Chongqing), (8)Chifeng Municipal Hospital (Neimenggu Province), and (9)the first affiliated hospital of Jilin University (Jilin Province).The LC patients with diverse geographic distribution and socioeconomic backgrounds were all enrolled in **P**rospective su**R**veillance for very **E**arly hepato**C**ellular c**AR**cinoma project (PreCar, funded by the National Natural Science Foundation of China and others; registered in the WHO International Clinical Trial Registry Platform, ClinicalTrials.gov number, NCT03588442). 490 healthy participants were recruited for constructing the negative background pool. Blood samples from all individuals were collected to extract cfDNA and conduct cSMART assay. Of the 1185 participants, 689 (344 LC and 345 HCC), 296 (145 LC and 151 HCC), and 200 (88 LC and 112 HCC) were randomly grouped into training, test, and validation cohort to develop diagnostic method for HCC and evaluate the performance of the novel model, respectively. Neither the patient, nor the physician knew the group to which the participant be-longed (i.e., this component was double-blinded). Our sequencing data were available for replication or reuse by other researchers.

**Participants**

We recruited 1675 individuals in this study. Population selection and attrition of our study were shown in Figure 1. For the 608 HCC patients (diagnosed as HCC pathologically), 85.53% were males, 53.78% were 40-60 years old, 87.00% were diagnosed with HBV infection, and the proportions of HCC patients with Child-Pugh scores 0/A, B, and C were 96.86%, 2.97%, and 0.17%, respectively. For the 529 LC patients, 71.75% were males, 68.28% were 40-60 years old, 81.95% were diagnosed with HBV infection, and liver function Child-Pugh classification A, B, and C accounted for 83.11%, 16.51%, and 0.38%, respectively (Table S1). Other basic information of enrolled patients such as age, AFP, AFP-L3, and PIVKA II were analyzed and displayed in Table 1 and Table S1. HCC were confirmed by histopathology according to the American Association for the Study of Liver Diseases (AASLD) guidelines[1]. The inclusion criteria of LC was as follows: 1) Metavir score of 4 or Ishak score of 5 to 61; 2) ascites, hepatic encephalopathy, or variceal hemorrhage, excluding those caused by reasons other than liver cirrhosis; 3) satisfying at least 2 of below conditions: Imaging indicating characteristics of liver cirrhosis, platelet count<200×10^9^/L, liver stiffness measurements >12 kPa when ALT <5 folds upper limit of normal[2]. The degree of liver failure was determined by Child-Pugh score[3]. The research protocol was reviewed and approved by ethics committee at all participating hospitals. A written informed consent was provided by every participant.

**Blood test**

10mL blood of each participant was collected in Cell-Free DNA BCT streck tubes and sent to National Center for Liver Cancer for plasma separation. Plasma was separated by two rounds of centrifugation, and the plasma samples were then transported to Berry Genomics Corporation for cfDNA extraction (MagMAX Cell-Free DNA isolation Kit (Thermo Fisher Scientific)) and cSMART assays[4, 5].

**cSMART assay and Negative background pool construction**

cSMART assay was conducted using cfDNA samples from 490 healthy individuals, 577 LC patients, and 608 HCC patients. 10 ng cfDNA of each sample containing unique 6-bp barcodes was prepared for building libraries. Barcoded molecules were amplified through polymerase chain reaction (PCR), circularized, and reamplified with target-specific bidirectional back-to-back primers to replicate targeted alleles through inverse PCR. Primers were designed targeting 931 regions among 21 genes. The products of this kind of amplification preserved both barcodes and the length information of the original cfDNA molecules. NextSeq500 platform (Illumina, San Diego, CA, USA) was applied to perform high-depth sequencing on the inversely amplified products (ligated with Illumina TruSeq adapter), and paired-end reads of 2×200 bp were generated. Unique single barcoded molecules from the preamplification library were counted and the final mutant allelic ratio was determined. Detailed information of the cSMART assay were as described in previous studies[4, 6, 7].

Next, rules were formulated to determine the positive gene mutations. We first constructed the negative background pool with cfDNA samples from 490 healthy individuals using binomial distribution according to the following formula:

$$p=\frac{t!}{m!\left( t-m \right)!}\left( \frac{\sum_{j=1}^{n} m_{i}^{j}}{\sum_{j=1}^{n} t_{i}^{j}} \right)^{m}\left( 1-\frac{\sum_{j=1}^{n} m_{i}^{j}}{\sum_{j=1}^{n} t_{i}^{j}} \right)^{t-m}$$

(n: number of the negative samples, $m_{i}^{j}$: number of mutation templates covering gene site i in negative samples, $t_{i}^{j}$: number of templates covering site i in negative sample j, m: number of mutation templates containing target gene site in the samples to be tested, t: number of templates containing target sites in the samples to be tested).

Then positive mutations were identified if the number of mutation templates at this gene site was not less than one as well as the hypothesis that the gene site was negative was rejected (P < 0.01).

**Model construction**

Considering the cost-effectiveness and feasibility of the novel diagnostic test, we minimize the number of detected mutations while ensuring a sufficiently high diagnostic sensitivity by optimizing the screening process according to the following rules: 1. P value < 0.05 (calculated by Fisher exact test), 2. Less than 10 LC samples were positive at the specific mutation site, 3. More than 10 HCC samples were positive at the specific mutation site. Applying these rules on the cSMART assay data of the training cohort, three mutation sites were screened out to be further analysis. These three mutation sites located in different regions of gene TERT, TP3, and CTNNB1. The machine learning analysis identified four clinical variables as risk factors: AFP，AFP-L3 ,PIVKA-Ⅱ, and genetic mutations of cfDNA. These four clinical variables were used as the input of the machine learning models. AFP，AFP-L3 , and PIVKA-Ⅱ were converted into 0 or 1 according to their respective cutoff values. The three mutation sites located in gene TP53, TERT, and CTNNB1 were identified as positive or negative according to the specified rules. As long as at least one of them was positive, input 1 for this parameter, otherwise input 0. The modeling process was performed as follow: the input was the four clinical variables transformed by 0 and 1; the output was a determination of whether a subject has developed HCC.

In this study, Extreme Gradient boosting (XGBoost) was used to established a diagnostic model for HCC. The XGBoost algorithm was executed using ‘Numpy’, ‘Scikit Learn’, ‘matplotlib’, and ‘XGBoost’ packages in Python software. In order to avoid overfitting and improve the accuracy of the machine learning model, 10-fold cross-validation was adopted to determine the optimal parameters, and the best model was applied to the test cohort to evaluate the diagnostic performance of the model. Finally, the probability value with the largest Youden index was determined as the optimal threshold value (0.54), and then an independent external validation cohort were introduced to further verify the performance of the model.

**Statistical analysis**

Wilcoxon rank-sum test and Kruskal-Wallis rank-sum test were used to compare the differences between two or three groups of continuous variables, respectively. Chi-square test and Fisher’s exact test were applied to determine the differences in categorical variables. P value was calculated using Python software, version 2.7.14, and P<0.05 was considered as statistically significant.

# References

1. Heimbach JK, Kulik LM, Finn RS et al. AASLD guidelines for the treatment of hepatocellular carcinoma. Hepatology 2018; 67: 358-380.

2. Chen L, Abou-Alfa GK, Zheng B et al. Genome-scale profiling of circulating cell-free DNA signatures for early detection of hepatocellular carcinoma in cirrhotic patients. Cell Res 2021; 31: 589-592.

3. Krens SD, Lassche G, Jansman FGA et al. Use of the Child-Pugh score in anticancer drug dosing decision making: proceed with caution - Authors' reply. Lancet Oncol 2019; 20: e290.

4. Lv W, Wei X, Guo R et al. Noninvasive prenatal testing for Wilson disease by use of circulating single-molecule amplification and resequencing technology (cSMART). Clin Chem 2015; 61: 172-181.

5. Zheng B, Liu XL, Fan R et al. The Landscape of Cell-Free HBV Integrations and Mutations in Cirrhosis and Hepatocellular Carcinoma Patients. Clin Cancer Res 2021; 27: 3772-3783.

6. Wang Z, Cheng G, Han X et al. Application of Single-Molecule Amplification and Resequencing Technology for Broad Surveillance of Plasma Mutations in Patients with Advanced Lung Adenocarcinoma. J Mol Diagn 2017; 19: 169-181.

7. Chen K, Zhao H, Shi Y et al. Perioperative Dynamic Changes in Circulating Tumor DNA in Patients with Lung Cancer (DYNAMIC). Clin Cancer Res 2019; 25: 7058-7067.

**Table S1. Basic information of enrolled patients**

|  |  | **HCC** | | | | |  | **LC** | | | | |
| --- | --- | --- | --- | --- | --- | --- | --- | --- | --- | --- | --- | --- |
|  | **Value** | **All**  **(n=608)** | **Training**  **(n=345)** | **Test**  **(n=151)** | **Validation**  **(n=112)** | **P** |  | **All**  **(n=577)** | **Training**  **(n=344)** | **Test**  **(n=145)** | **Validation**  **(n=88)** | **P** |
| **Age** | <40 | 37 | 22 | 5 | 10 | 0.67 |  | 62 | 35 | 15 | 12 | 0.33 |
|  | 40-60 | 327 | 176 | 91 | 60 |  |  | 394 | 240 | 94 | 60 |  |
|  | >60 | 244 | 147 | 55 | 42 |  |  | 121 | 69 | 36 | 16 |  |
| **Gender** | Male | 520 | 289 | 132 | 99 | 0.36 |  | 414 | 246 | 101 | 67 | 0.56 |
|  | Female | 88 | 56 | 19 | 13 |  |  | 163 | 98 | 44 | 21 |  |
| **HBV** | Yes | 522 | 296 | 128 | 98 | 0.74 |  | 454 | 271 | 113 | 70 | 0.91 |
|  | No | 78 | 45 | 21 | 12 |  |  | 100 | 58 | 27 | 15 |  |
| **AFP** | <20 | 291 | 167 | 66 | 58 | 0.49 |  | 518 | 304 | 132 | 82 | 0.81 |
|  | 20-400 | 156 | 86 | 42 | 28 |  |  | 49 | 33 | 11 | 5 |  |
|  | >=400 | 161 | 92 | 43 | 26 |  |  | 10 | 7 | 2 | 1 |  |
| **AFP-L3** | <10% | 356 | 197 | 84 | 75 | 0.39 |  | 540 | 323 | 134 | 83 | 0.74 |
|  | >=10% | 252 | 148 | 67 | 37 |  |  | 37 | 21 | 11 | 5 |  |
| **PIVKA-**  **II** | <40 | 188 | 116 | 40 | 32 | 0.89 |  | 509 | 306 | 127 | 76 | 0.73 |
|  | >=40 | 420 | 229 | 111 | 80 |  |  | 68 | 38 | 18 | 12 |  |
| **CA199** | <37 | 516 | 292 | 128 | 96 | 0.73 |  | 394 | 226 | 105 | 63 | 0.28 |
|  | >=37 | 86 | 49 | 21 | 16 |  |  | 85 | 54 | 22 | 9 |  |
| **PLT** | <125 | 201 | 120 | 48 | 33 | 0.84 |  | 315 | 189 | 82 | 44 | 0.24 |
|  | 125-350 | 390 | 215 | 96 | 79 |  |  | 234 | 136 | 56 | 42 |  |
|  | >350 | 13 | 6 | 7 | 0 |  |  | 2 | 1 | 0 | 1 |  |
| **Child-**  **Pugh** | 0/A | 587 | 331 | 146 | 110 | 0.84 |  | 443 | 262 | 103 | 78 | 0.11 |
|  | B | 18 | 11 | 5 | 2 |  |  | 88 | 49 | 30 | 9 |  |
|  | C | 1 | 1 | 0 | 0 |  |  | 2 | 2 | 0 | 0 |  |
| **Tumor**  **size**  **(cm)** | <3 | 107 | 70 | 21 | 16 | 0.84 |  | - | - | - | - | - |
|  | 3-5 | 179 | 87 | 52 | 40 |  |  | - | - | - | - |  |
|  | 5-10 | 210 | 119 | 53 | 38 |  |  | - | - | - | - |  |
|  | >10 | 108 | 67 | 23 | 18 |  |  | - | - | - | - |  |
| **Tumor**  **count** | 1 | 505 | 287 | 123 | 95 | 0.77 |  | - | - | - | - | - |
|  | >1 | 103 | 58 | 28 | 17 |  |  | - | - | - | - |  |
| **BCLC**  **stage** | 0 | 36 | 24 | 5 | 7 | 0.83 |  | - | - | - | - | - |
|  | A | 114 | 64 | 30 | 20 |  |  | - | - | - | - |  |
|  | B | 151 | 87 | 36 | 28 |  |  | - | - | - | - |  |
|  | C | 306 | 170 | 79 | 57 |  |  | - | - | - | - |  |

**Table S2. Brief summary of all participants**

|  | **value** | **All(n=1185)**  n (%) | **HCC(n=608)**  n (%) | **LC(n=577)**  n (%) |
| --- | --- | --- | --- | --- |
| **Age** | <40 | 99(8.35%) | 37(6.09%) | 62(10.75%) |
|  | 40-60 | 721(60.84%) | 327(53.78%) | 394(68.28%) |
|  | >60 | 365(30.80%) | 244(40.13%) | 121(20.97%) |
| **Gender** | Female | 251(21.18%) | 88(14.47%) | 163(28.25%) |
|  | Male | 934(78.82%) | 520(85.53%) | 414(71.75%) |
| **HBV infection** | No | 178(15.42%) | 78(12.83%) | 100(18.05%) |
|  | Yes | 976(84.58%) | 522(87.00%) | 454(81.95%) |
| **AFP(ng/mL)** | <20 | 809(68.27%) | 291(47.86%) | 518(89.77%) |
|  | 20-400 | 205(17.30%) | 156(25.66%) | 49(8.49%) |
|  | >400 | 171(14.43%) | 161(26.48%) | 10(1.73%) |
| **PIVKA-II**  **(mAU/mL)** | <40 | 697(58.82%) | 188(30.92%) | 509(88.21%) |
|  | >=40 | 488(41.18%) | 420(69.08%) | 68(11.79%) |
| **CA199(U/mL)** | <37 | 910(84.18%) | 516(85.71%) | 394(82.25%) |
|  | >=37 | 171(15.82%) | 86(14.29%) | 85(17.75%) |
| **PLT(K/mL)** | <125 | 516(44.68%) | 201(33.28%) | 315(57.17%) |
|  | 125-350 | 624(54.03%) | 390(64.57%) | 234(42.47%) |
|  | >350 | 15(1.29%) | 13(2.15%) | 2(0.36%) |
| **TBIL(μmol/L))** | 1 | 1068(90.43%) | 593(97.53%) | 475(82.90%) |
|  | 2 | 61(5.17%) | 9(1.48%) | 52(9.08%) |
|  | 3 | 52(4.40%) | 6(0.99%) | 46(8.03%) |
| **ALB(g/L)** | 1 | 1061(89.84%) | 577(94.90%) | 484(84.47%) |
|  | 2 | 107(9.06%) | 27(4.44%) | 80(13.96%) |
|  | 3 | 13(1.10%) | 4(0.66%) | 9(1.57%) |
| **Child-Pugh stage** | 0/A | 1030(90.43%) | 587(96.86%) | 443(83.11%) |
|  | B | 106(9.31%) | 18(2.97%) | 88(16.51%) |
|  | C | 3(0.26%) | 1(0.17%) | 2(0.38%) |

**Table S13. Performance of single mutation site in the diagnosis of HCC**

| **Mutation**  **site** | **Test cohort（151HCC+145LC）** | | |  | **Validation cohort（112HCC+88LC）** | | |  |
| --- | --- | --- | --- | --- | --- | --- | --- | --- |
|  | **Sensitivity（95%CI）** | **Specificity（95%CI）** | **PPV（95%CI）** | **NPV（95%CI）** | **Sensitivity（95%CI）** | **Specificity（95%CI）** | **PPV（95%CI）** | **NPV（95%CI）** |
| **chr5_1295228** | 21.19 (15.14-28.74) | 100.00 (96.78-100.00) | 100.00 (86.66-100.00) | 54.92 (48.71%-60.99) | 16.96 (10.78-25.48) | 100.00 (94.79-100.00) | 100.00 (79.08-100.00) | 48.62 (41.17-56.12) |
| **chr17_7577534** | 10.60 (6.37-16.91) | 98.62 (94.60%-99.76) | 88.89 (63.93-98.05) | 51.44 (45.41-57.43) | 11.61 (6.57-19.37) | 100.00 (94.79-100) | 100.00 (71.66-100.00) | 47.06 (39.78-54.46) |
| **chr3_41266124** | 2.65 (0.85-7.07) | 100.00 (96.78-100.00) | 100.00 (39.58-100.00) | 49.66 (43.80-55.53) | 1.82 (0.31-6.94) | 100.00 (94.79-100.00) | 100.00 (19.79-100.00) | 44.44 (37.45-51.66) |


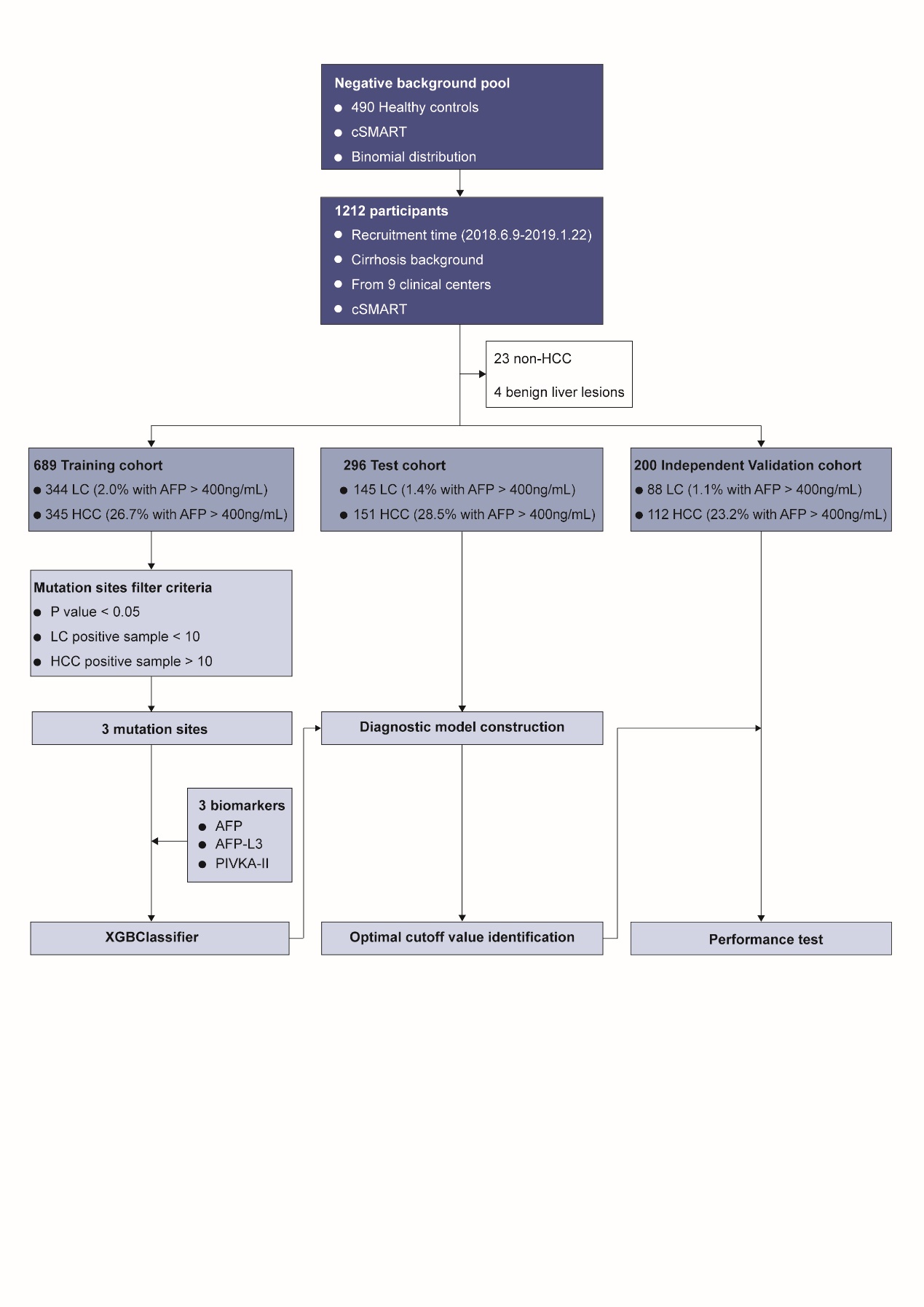


**Fig. S1.** **Study design**

cSMART: circulating single-molecule amplification and resequencing technology; LC: liver cirrhosis; HCC: hepatocellular carcinoma; AFP: alpha-fetoprotein; AFP-L3: alpha-fetoprotein lens culinaris agglutinin 3; PIVKA-Ⅱ: protein induced by vitamin K absence or antagonist-II.


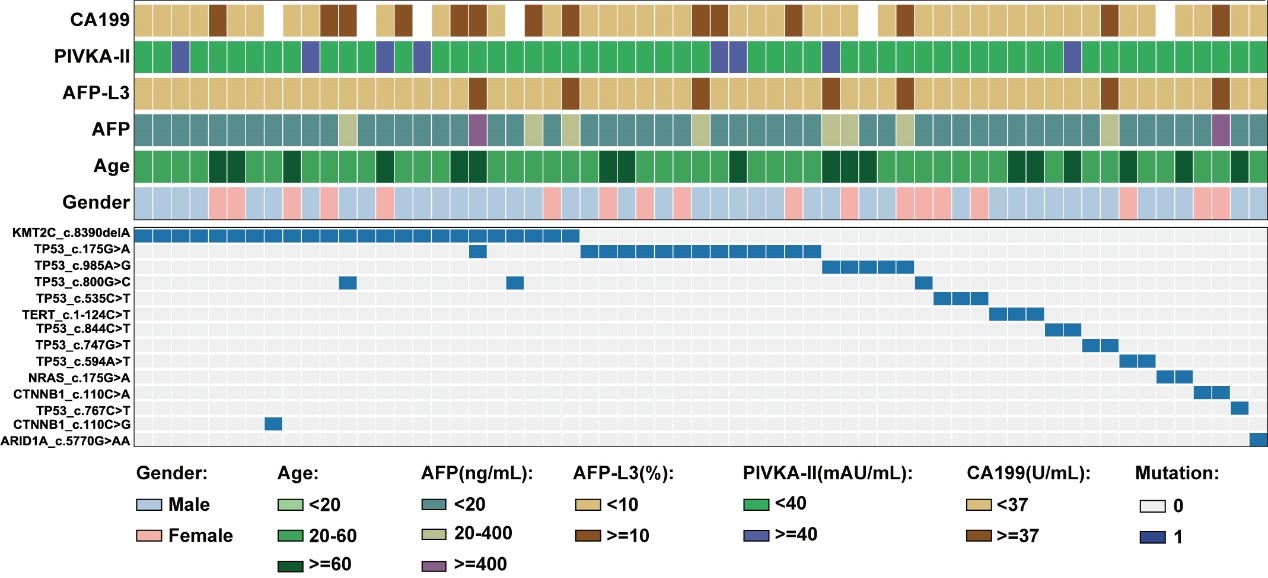


**Fig. S2. Clinical characteristics of LC patients with positive mutations at the top 20 high-frequency mutation sites.**

Basic information (age, gender), cirrhosis background, tumor serological biomarkers (CA199, PIVKA-II, AFP-L3, AFP), and HCC related parameters (MVI and BCLC stages) of all LC samples with positive mutations at the top 20 high-frequency mutation sites.


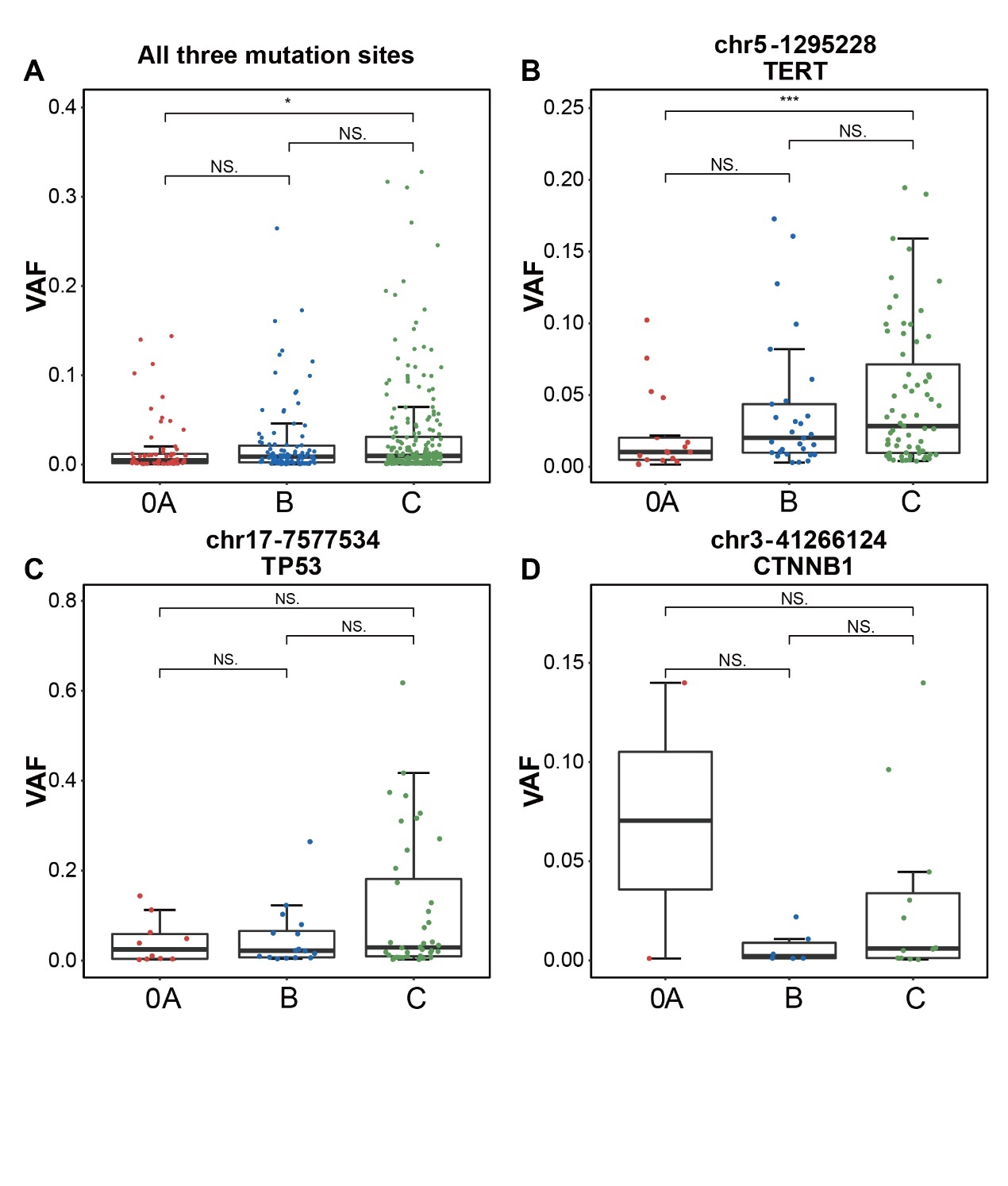


**Fig. S3. VAF of** **HCC-specific high-frequency mutation sites were closely related to the stage of HCC.**

(A) The overall VAFs of three HCC-specific high-frequency mutation sites (Chr5-1295228 TERT, Chr17-7577534 TP53, and Chr3-41266124 CTNNB1) in different stages of HCC samples. VAF: variant allele frequency; Chr: chromosome. *P<0.05, **P<0.01, ***P<0.001, Wilcoxon rank-sum test. (B) The overall VAFs of HCC-specific high-frequency mutation site Chr5-1295228 TERT in different stages of HCC samples. *P<0.05, **P<0.01, ***P<0.001, Wilcoxon rank-sum test. (C) The overall VAFs of HCC-specific high-frequency mutation site Chr17-7577534 TP53 in different stages of HCC samples. *P<0.05, **P<0.01, ***P<0.001, Wilcoxon rank-sum test. (D) The overall VAFs of HCC-specific high-frequency mutation site Chr3-41266124 CTNNB1 in different stages of HCC samples. *P<0.05, **P<0.01, ***P<0.001, Wilcoxon rank-sum test.


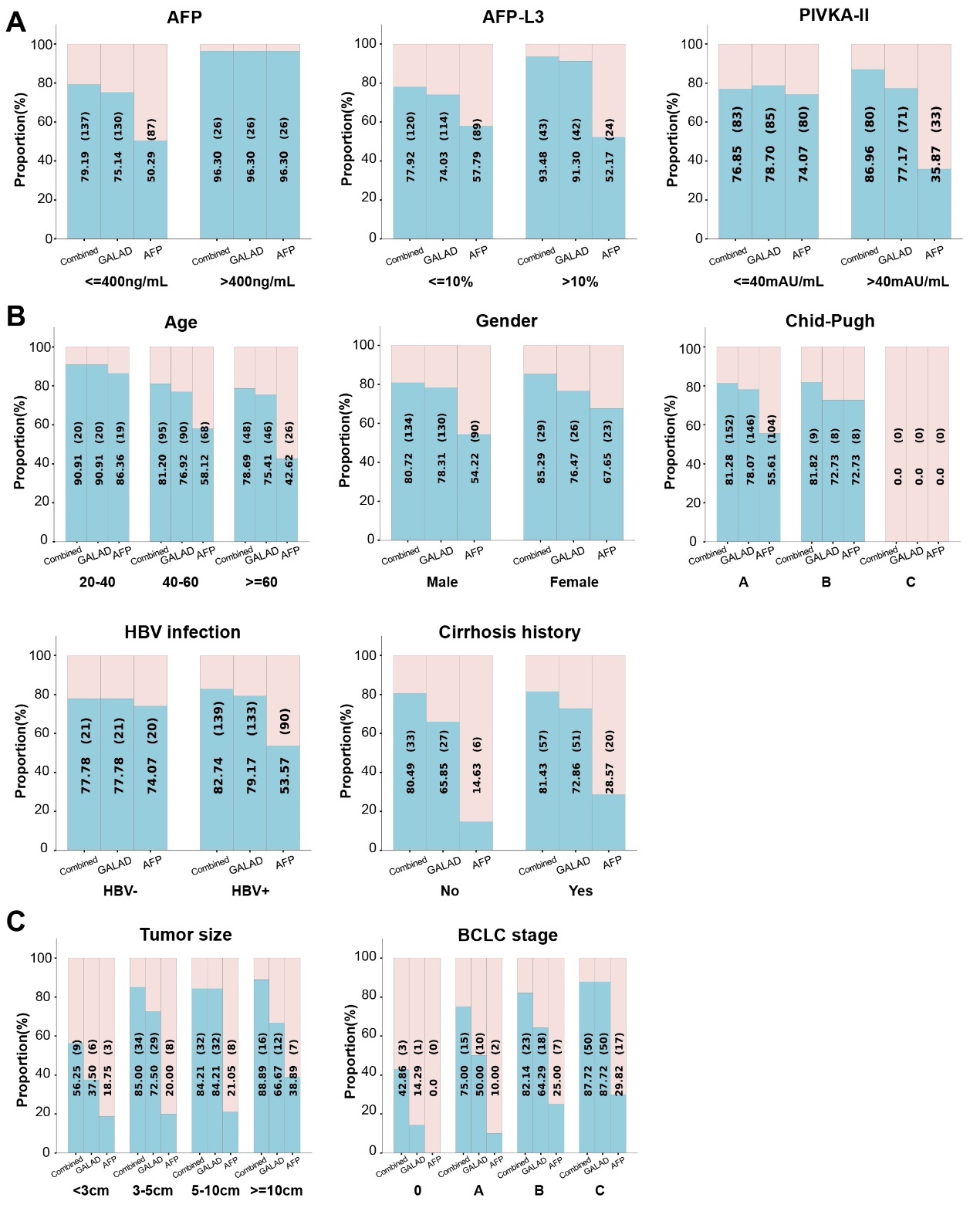


**Fig. S4. The diagnostic performance of Combined model in different subgroups of the validation cohort.**

(A) Proportions of positive and negative calling by Combined method GALAD and AFP in all participants with different AFP, AFP-L3 and PIVKA-II levels in validation cohort. (B) Proportions of positive and negative calling by Combined method, GALAD and AFP in all participants with different age, gender, Child-Pugh stages, HBV infection status and cirrhosis history in validation cohort. (C) Proportions of positive and negative calling by Combined method, GALAD and AFP in HCC patients with different tumor sizes and BCLC stages in validation cohort.
